# Effectiveness of a single COVID-19 mRNA vaccine dose in individuals with prior SARS-CoV-2 infection: a systematic review

**DOI:** 10.1038/s43856-025-00882-y

**Published:** 2025-05-03

**Authors:** Hannah R. Volkman, Jennifer L. Nguyen, Mustapha M. Mustapha, Jingyan Yang, Luis Jodar, John M. McLaughlin

**Affiliations:** 1 Pfizer Inc., New York, NY USA; 2 Department of Population Health, Hofstra University, Hempstead, NY USA; 3 Institute for Social and Economic Research and Policy, Columbia University, New York, NY USA

**Keywords:** Viral infection, Epidemiology, RNA vaccines

## Abstract

**Background:**

Based on high population immunity to SARS-CoV-2 from prior infection, vaccination, or both, in fall 2023, regulatory agencies globally authorized/approved a single mRNA XBB.1.5-adapted vaccine dose for individuals aged ≥5 years regardless of prior vaccination.

**Methods:**

We conducted a systematic review on vaccine effectiveness (VE) of a single COVID-19 mRNA dose in individuals with a history of prior infection compared to individuals who were (i) SARS-CoV-2 naïve, (ii) unvaccinated with prior infection, and (iii) vaccinated with >1 dose with or without prior infection. We searched MEDLINE and Embase for studies published January 2021–October 2023. Data were synthesized following Synthesis Without Meta-Analysis guidelines; bias was assessed using the Newcastle-Ottawa Scale. This study was registered with PROSPERO (CRD42023453257).

**Results:**

Eighteen studies were eligible. None of these studies reported bivalent or XBB.1.5-adapted VE, and none reported VE for immunocompromised populations or children aged <5 years. Among those with prior infection, a single mRNA dose increased protection by 8–71% against infection (during Omicron BA.1, BA.4/5, or XBB predominance), 39–67% against symptomatic infection (BA.1, BA.2, or BA.4/5), and 25–60% against hospitalization or hospitalization or death (BA.1). VE of one dose was comparable to two doses among those with prior infection, and higher than following two doses without prior infection.

**Conclusions:**

A single dose of original mRNA COVID-19 vaccine provides similar protection to two doses for immunocompetent individuals aged ≥5 years in the current setting of high pre-existing immunity. This supports current recommendations for one dose to be given in advance of the respiratory season, regardless of history of infection or vaccination, with considerations for additional doses for certain populations including young children, older adults, and the immunocompromised.

## Plain language summary

We reviewed the existing literature on the effectiveness a single dose of a COVID-19 mRNA vaccine among people who have been exposed the virus that causes COVID-19 (SARS-CoV-2) in the past. We found that a single vaccine dose provides significant added protection above that gained from a past SARS-CoV-2 infection alone. Among people with a prior history of infection, there was no difference in the level of protection between one or two subsequent COVID-19 vaccine doses. This evidence supports recommendations that a single dose of COVID-19 mRNA vaccine given at the start of the cold and flu season provides significant protection from COVID-19, regardless of past illnesses.

## Abstract (web summary)

Volkman et al. review the evidence on whether a single COVID-19 mRNA vaccine dose is effective against future infections. A single dose provides significant added protection above that gained from a prior SARS-CoV-2 infection alone, and there was no significant difference between a single or two doses in those previously infected with SARS-CoV-2.

## Introduction

Since the emergence of Omicron in November 2021, population-level immunity to SARS-CoV-2 stemming from both natural infection and vaccination has increased substantially. Recent seroprevalence studies conducted in the United States, Canada, United Kingdom, and Europe estimate that >90% of the population have some immunity to SARS-CoV-2 from prior infection, vaccination, or both^1–4^. However, due to the emergence of antigenically distinct variants and waning immunity^5,6^, populations remain at risk for future infection, severe disease, and sequelae of SARS-CoV-2 infections.

COVID-19 mRNA vaccines were initially licensed as a two-dose series, with subsequent approvals, and recommendations for additional booster doses for various populations during the pandemic. Based on the high level of population immunity to SARS-CoV-2 and in support of simplifying the vaccine dosing schedule, regulatory authorities have since authorized or approved the use of a single updated COVID-19 mRNA dose. In the United States, the Food and Drug Administration authorized or approved the use of a single BA.4/5 bivalent COVID-19 mRNA dose for most individuals above 5 years of age on April 18, 2023^7^. This was followed by authorization of a single annual dose of monovalent COVID-19 mRNA XBB.1.5-adapted vaccine for the same age group on September 11, 2023 intended for primary vaccination and for the 2023–2024 respiratory virus season^8^. In a similar manner, the European Medicines Agency authorized or approved a single dose of the BNT162b2 XBB.1.5-adapted vaccine on August 30, 2023 for individuals ≥5 years of age irrespective of prior COVID-19 doses^9^. Likewise, the World Health Organization Strategic Advisory Group of Experts issued a recommendation on September 28, 2023 for a single annual dose for primary immunization citing potential benefits in terms of improved vaccine acceptance and uptake^10^.

Considering these updated COVID-19 vaccination guidelines, it is necessary to better understand the level of protection provided by a single mRNA vaccine dose in the current immunological context of high population-level immunity. The aim of this systematic review was to summarize the peer-reviewed literature on vaccine effectiveness (VE) of a single COVID-19 mRNA dose among individuals with a history of prior SARS-CoV-2 infection, compared to those with differing prior infection and vaccination history, including individuals who were (i) SARS-CoV-2 naïve (i.e., unvaccinated and no history of prior infection), (ii) unvaccinated with prior SARS-CoV-2 infection, and (iii) previously vaccinated with >1 COVID-19 dose with or without prior infection.

This systematic review identifies 18 studies and finds that among those with prior infection, a single mRNA dose increases protection by 8–71% against infection (during Omicron BA.1, BA.4/5, or XBB predominance), 39–67% against symptomatic infection (BA.1, BA.2, or BA.4/5), and 25–60% against hospitalization or hospitalization or death (BA.1). VE of one dose is comparable to two doses among those with prior infection, and higher than following two doses without prior infection. This supports current recommendations for one COVID-19 mRNA vaccine dose to be given in advance of the respiratory season, regardless of history of infection or vaccination, with considerations for additional doses for certain populations including young children, older adults, and the immunocompromised.

## Methods

### Search strategy and selection criteria

We conducted this systematic literature review according to Preferred Reporting Items for Systematic Reviews and Meta-Analyses (PRISMA)^11^ and Synthesis Without Meta-Analysis (SWiM) guidelines^12^. The protocol was registered with PROSPERO (CRD42023453257). We searched MEDLINE and Embase using Ovid for case-control and cohort studies reporting VE for one dose of COVID-19 mRNA vaccine among individuals of any age and immunocompetence status with prior SARS-CoV-2 infection published from January 1, 2021 to October 4, 2023. The search strategy included two concepts: COVID-19 mRNA vaccines and prior SARS-CoV-2 infection (Supplementary Table 1).

### Data synthesis and analysis

**Fig. 1:**
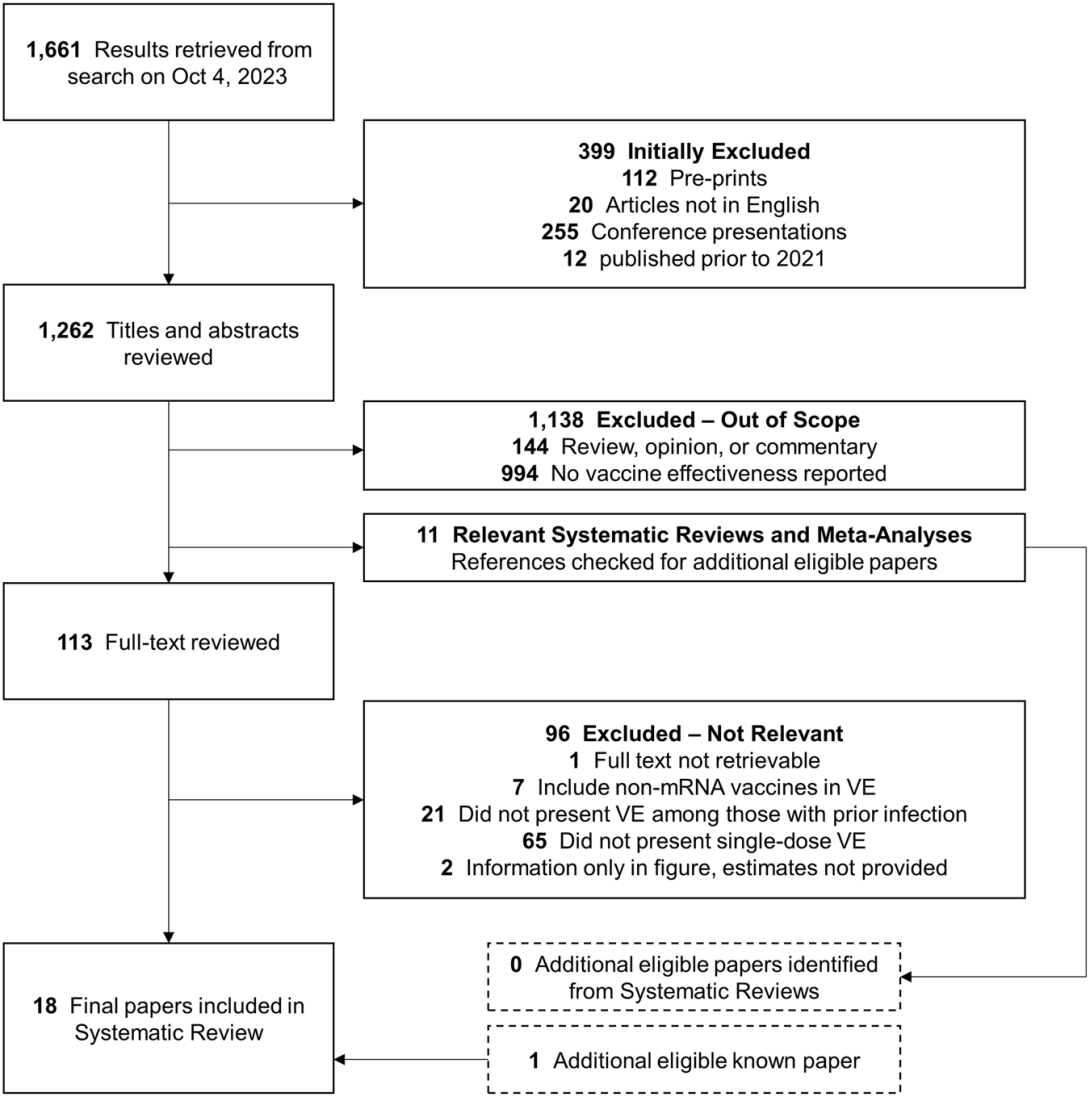
PRISMA flow chart. The number of studies identified from the systematic review search strategy are depicted with counts of studies excluded and the reason for exclusion at each round of the review process.

Pre-prints, articles not in English, conference presentations, and papers published before 2021 were excluded (Fig. 1). Study titles and abstracts were independently screened by two investigators to identify studies reporting COVID-19 VE. Papers were excluded if they were reviews or opinions, clinical, interventional, or laboratory studies, or if VE was not reported. Relevant systematic reviews and meta-analyses were reviewed to identify potential additional studies missed by the search. Full text and supplements were then reviewed for remaining articles by both investigators with disagreements resolved by a third investigator. Studies were excluded if any of following criteria were met: the full text was not retrievable, non-mRNA vaccines were included in VE estimates, VE was not reported for individuals with prior infection, single dose VE was not presented, or VE point estimates and 95% confidence intervals (95% CI) could not be determined (e.g., estimates provided in a figure only), which happened infrequently.

We extracted information on study population, VE, comparator groups (i.e., (i) SARS-CoV-2 naïve, (ii) unvaccinated with prior SARS-CoV-2 infection, and (iii) vaccinated with >1 dose with or without prior infection), sample size, age group, predominant circulating variant during the study period, and other factors from eligible studies (Supplementary Table 2). When VE estimates were stratified by time since dose, we extracted the earliest estimate reported at ≥14 days after vaccination, regardless of dose number. For studies conducted during the Omicron period where authors did not report predominant subvariant(s), we used country-specific data from CoVariants^13^ to assign a subvariant. Other missing information was noted and not imputed. VE estimates were extracted as reported in each study without consolidation of stratified data (Supplementary Tables 3–5). No duplicate data were included. The main outcome was COVID-19 mRNA VE point estimates and 95% CIs among individuals with prior infection and receipt of a single vaccine dose compared to any other group. For studies that did not report VE directly but instead provided the hazard ratio, odds ratio, or relative risk ratio, we calculated the VE as (1 – adjusted ratio) × 100%^14^. When comparing multiple VE estimates from a single study, estimates without overlapping 95% CIs were considered significantly different. VE estimates were summarized as ranges of the lowest and highest VE point estimates for studies in a group. VE against infection was defined as effectiveness against asymptomatic or symptomatic infection.

**Fig. 2:**
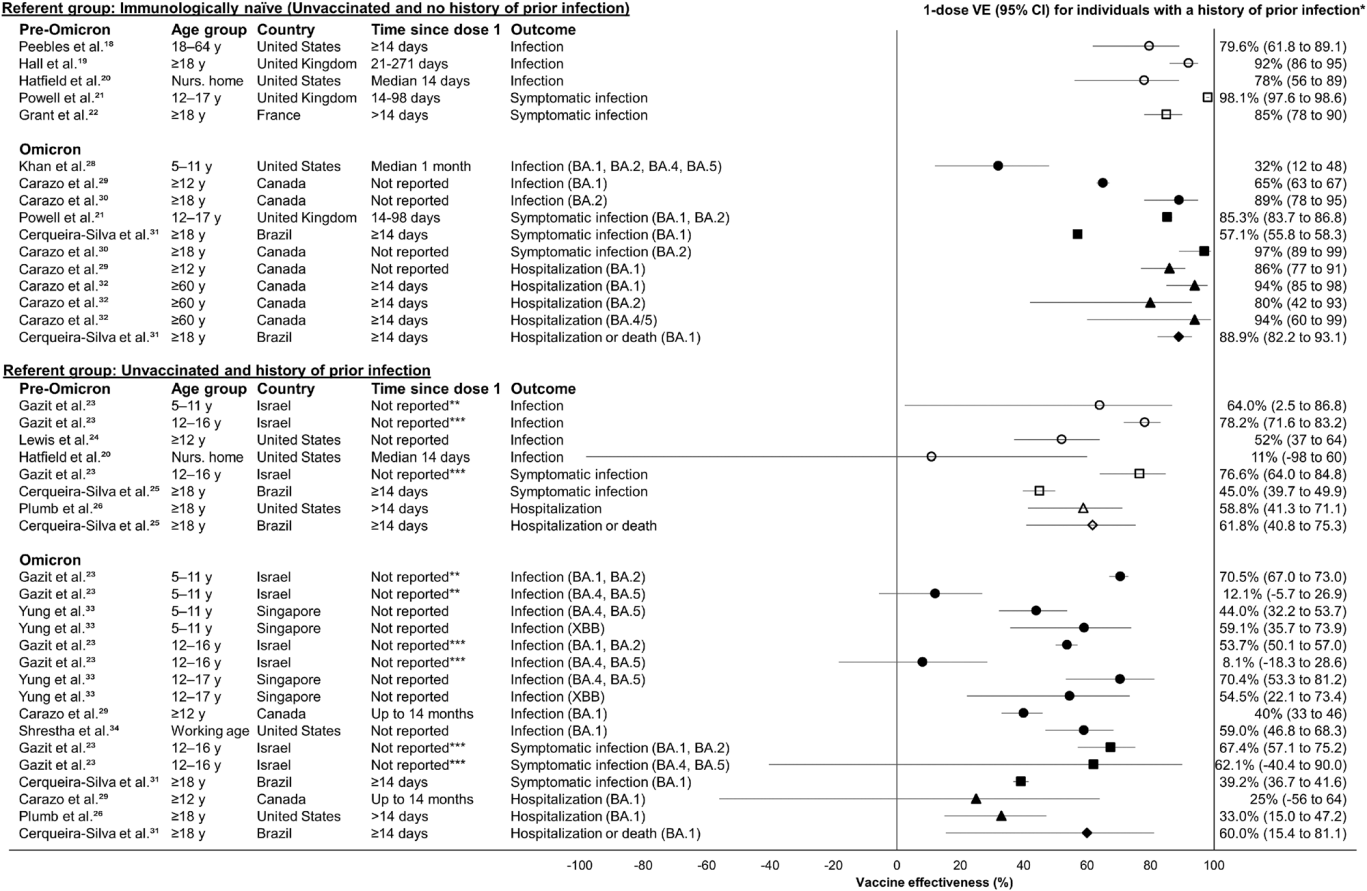
Forest plot of studies reporting single dose COVID-19 mRNA vaccine effectiveness estimates for immunocompetent individuals with a history of prior infection. Hollow shapes represent vaccine effectiveness (VE) during pre-Omicron predominance. Solid shapes represent VE during Omicron predominance. Circles represent VE against infection; squares represent VE against symptomatic infection; triangles represent VE against hospitalization; diamonds represent VE against hospitalization or death. *VE and 95% CI values are shown with significant digits as presented by the original study authors. **Gazit et al.^23^ reports mean follow-up time for 5–11-year-olds as: 161 days among vaccinated and 86 days among unvaccinated participants. *** Gazit et al.^23^ reports mean follow-up time for 12–16-year-olds as: 244 days among vaccinated and 114 days among unvaccinated participants.

Groups were defined for synthesis prior to data extraction in the following order, from highest to lowest priority: referent group of the VE estimate, variant period (i.e., pre-Omicron, Omicron), VE outcome, age group at vaccination, and predominant sub-variant during study follow-up (Supplementary Fig. 1). Groups were defined since VE estimates cannot meaningfully be synthesized across these groups. All VE outcomes presented in eligible studies were extracted for all permutations of synthesis group. VE estimates for other strata, including non-mRNA vaccines, and order of infection and vaccine dose(s) were considered out of scope and not extracted. During data extraction, studies were organized according to the five pre-defined groups and no additional synthesis groups were identified based on study characteristics. To informally examine heterogeneity, studies were ordered in Fig. 2 and Supplementary Tables 3–5 by the groups described above.

### Study quality assessment

We performed a bias assessment for all eligible studies using the Newcastle-Ottawa Quality Assessment Scale for case-control and cohort studies^15^, (Supplementary Tables 6–7). Seven or more stars was set as the quality threshold below which papers would be excluded. Two investigators conducted the bias assessment with disagreements resolved by a third investigator. VE estimates with 95% CIs spanning ≥50 percentage points are noted in Supplementary Tables 3–5 due to low certainty of evidence. Results are reported to the nearest integer in the text and with the same number of significant digits as presented by the authors in figures and tables.

### Reporting summary

Further information on research design is available in the Nature Portfolio Reporting Summary linked to this article.

## Results

### Study selection

Of 1661 query results, 1262 were eligible for title and abstract screening, and 113 were eligible for full-text review, yielding 17 studies reporting single dose COVID-19 mRNA VE in populations with a history of prior infection (Supplementary Table 1). One study known to be eligible for inclusion but not identified in the search or included in reference lists of relevant systematic reviews was also included, for a total of 18 studies (Fig. 1). Two studies appeared to fit the inclusion criteria but single dose VE was reported only in figures without corresponding values for point estimates and 95% CIs and thus were excluded^16,17^.

### Study characteristics

The 18 included studies encompassed over 5.8 million total patients (Supplementary Table 2). Studies were conducted in the United States (6), Canada (3), Brazil (2), Israel (2), United Kingdom (2), China (Hong Kong [1]), France (1), and Singapore (1). Study designs included eight test-negative case-control, one matched case-control, eight retrospective cohort, and one prospective cohort. All included studies reported single dose VE for original mRNA vaccines, and no studies reported single dose VE for BA.1 or BA.4/5 bivalent or monovalent XBB.1.5-adapted formulations. VE endpoints reported in studies included infection, symptomatic infection, hospitalization, and hospitalization or death.

Ten studies included adult populations, of which five were conducted in high-risk populations including health system workers (3), nursing home residents (1), and adults ≥60 years (1). Four studies included both adolescent and adult populations (aged ≥12 years [3]; ≥16 years [1]). Three studies reported VE for adolescents aged 12–16 or 12–17 years, and three studies reported VE for children aged 5–11 years. No studies were identified reporting single dose VE for children under 5 years of age with prior infection. Ten studies reported VE during periods predominated by the original strain, or Alpha or Delta variants, while 11 studies reported VE during Omicron predominance. Ten studies reported single dose VE for the BNT162b2 vaccine alone while the remaining eight studies reported VE estimates for any mRNA vaccine (BNT162b2 or mRNA-1273).

### VE during pre-Omicron predominance

Ten studies conducted in the United States, United Kingdom, France, Israel, and Brazil reported single dose mRNA VE for individuals with history of prior infection during periods of wild-type, Alpha, or Delta strain predominance (Supplementary Table 2). These studies used three different referent groups: immunologically naïve (unvaccinated individuals with no history of prior infection [5 studies])^18–22^, unvaccinated individuals with a history of prior infection (5 studies)^20,23–26^, and individuals vaccinated with two doses with no history of prior infection (1 study, Supplementary Tables 3–5)^27^.

Compared to SARS-CoV-2 naïve individuals (i.e., unvaccinated individuals with no history of prior infection), one dose VE among individuals with history of prior infection ranged from 78 to 92% against infection^18–20^, and 85–98% against symptomatic infection during wild-type, Alpha, or Delta strain predominance^21,22^. In all five studies, single dose VE among those with prior infection was similar (i.e., had overlapping 95% CIs) to two dose VE among those with prior infection (Table 1); two dose VE with prior infection ranged from 78 to 85% against infection^18–20^, and 96 to 99% against symptomatic infection (Supplementary Table 3)^21,22^. Three studies found that effectiveness against infection with a single dose in individuals previously infected with SARS-CoV-2 was comparable to two vaccine doses without prior infection^18–20^. Two studies found that one dose plus prior infection afforded significantly higher protection against symptomatic infection than two vaccine doses without prior infection (Table 1)^21,22^.

**Table 1:** Prior infection and 1 dose vaccine effectiveness (VE) performance against various comparators during original, Alpha, and Delta (pre-Omicron) variant predominance among immunocompetent individuals

Comparator group		Prior infection and 1 dose VE is:		
Infection status	Vaccination status	Lower	Comparable	Higher
No infection	Unvaccinated	NE	NE	Infection^18–20^ Symptomatic infection^21,22^
Prior infection	Unvaccinated	NE	Infection^18–20^ Symptomatic infection^22^	Infection^23,24^ Symptomatic infection^21,23,25^ Hospitalization^25,26^
No infection	1 dose	NE	Infection^18^	Infection^19,20^ Symptomatic infection^21,22^
No infection	2 doses	NE	Infection^18–20^	Symptomatic infection^21,22^
No infection	3 doses (i.e., 2 primary doses + 1 booster)	NE	Symptomatic infection^21^	NE
Prior infection	2 doses	Symptomatic infection^25^	Infection^18–20,24,27^ Symptomatic infection^21,22^ Hospitalization^25,26^	NE
Prior infection	3 doses (i.e., 2 primary doses + 1 booster)	NE	Hospitalization^26^	NE

As an example for interpretation of the table, the first row below the header indicates that three studies (refs. ^18–20^) report that prior infection and 1 dose VE against infection is higher compared to no infection and no vaccination during pre-Omicron predominance. NE represents no evidence, signifying that no studies found that prior infection and one dose has comparable or lower VE compared to no infection and no vaccination.

Five studies reported one dose VE among individuals with history of prior infection compared to unvaccinated individuals with history of prior infection during wild-type, Alpha, and Delta predominance. One dose VE against infection ranged from 52 to 78% in study populations that included adolescents and adults^23,24^, and was lower (11%, 95% CI: −98 to 60) in a study of nursing home residents (Fig. 2)^20^. One dose VE was 45–77% against symptomatic infection^23,25^, 59% (95% CI: 41 to 71) against hospitalization^26^, and 62% (95% CI: 41 to 75) against hospitalization or death^25^. One dose without prior SARS-CoV-2 infection had lower VE than one dose with prior infection^19–22^, and two doses plus prior infection conferred similar^20,24–26^ or higher^25^ protection as one dose plus prior infection (Table 1, Supplementary Table 4).

In the only study that directly compared receipt of one dose with prior SARS-CoV-2 infection to two doses with prior infection as the referent group, there was no significant difference in VE for one or two doses with prior infection (Supplementary Table 5)^27^.

### VE during Omicron predominance

Eleven studies conducted in Brazil, Canada, China (Hong Kong), Israel, Singapore, the United States, and the United Kingdom reported single dose mRNA VE for individuals with history of prior infection during Omicron predominance. Six studies reported VE compared to immunologically naïve individuals^21,28–32^, and six studies reported VE compared to unvaccinated individuals with history prior infection^23,26,29,31,33,34^. One study used single dose plus prior infection as a referent group for estimating effectiveness of two or three doses plus prior infection^34^, and one study used two doses plus no prior infection as the singular referent group^35^.

Compared to immunologically naïve individuals, VE ranged from 32 to 89% against infection^28–30^, 57–97% against symptomatic infection^21,30,31^, 80–94% against hospitalization^29,32^, and was 89% (95% CI: 82 to 93) for the only estimate against hospitalization or death (Fig. 2)^31^. Effectiveness of a single dose with prior infection was comparable^28,29,32^ or higher than for one or two doses without prior infection against Omicron infection^29,30^, symptomatic infection^21,30^, and hospitalization (Table 2, Supplementary Table 3)^29,32^. A single dose with prior infection conferred similar protection as two doses with prior infection against all endpoints in 10 estimates^21,28–32^, with one estimate reporting higher effectiveness against symptomatic infection for two doses plus prior infection than one dose plus prior infection^31^.

**Table 2:** Prior infection and 1 dose vaccine effectiveness (VE) performance against various comparators during Omicron variant predominance among immunocompetent individuals

Comparator group		Prior infection and 1 dose VE is:		
Infection status	Vaccination status	Lower	Comparable	Higher
No infection	Unvaccinated	NE	NE	Infection^28–30^ Symptomatic infection^21,30,31^ Hospitalization^29,32^ Hospitalization or death^31^
Prior infection	Unvaccinated	NE	Infection^23,30^ Symptomatic infection^23,32^ Hospitalization^29,32^ Hospitalization or death^31^	Infection^23,29,33,34^ Symptomatic infection^21,23,31^ Hospitalization^26^ Hospitalization or death^31^
No infection	1 dose	NE	Infection^28^ Hospitalization^32^	Infection^29,30^ Symptomatic infection^21,30^ Hospitalization^29,32^
No infection	2 doses	NE	Infection^28^ Hospitalization^29,32,35^	Infection^29,30,35^ Symptomatic infection^21,30^ Hospitalization^32^
No infection	3 doses (i.e., 2 primary doses + 1 booster)	Infection^29^	Infection^28^ Hospitalization^29,32^	Infection^30^ Symptomatic infection^21,30^
Prior infection	2 doses	Infection^33^ Symptomatic infection^31^	Infection^28–30,33^ Symptomatic infection^21,30^ Hospitalization^26,29,32^ Hospitalization or death^31^	Infection^34^
Prior infection	3 doses (i.e., 2 primary doses + 1 booster)	Infection^28,29^ Symptomatic infection^31^ Hospitalization^26,29^	Infection^30,33,34^ Symptomatic infection^21,30^ Hospitalization^32^ Hospitalization or death^31^	NE

As an example for interpretation of the table, the first row below the header indicates that three studies (refs. ^28–30^) report that prior infection and 1 dose VE against infection is higher compared to no infection and no vaccination during Omicron predominance. NE represents no evidence, signifying that no studies found that prior infection and one dose has comparable or lower VE compared to no infection and no vaccination.

Compared to unvaccinated individuals with prior infection, VE of one dose plus prior infection ranged from 8 to 71% against infection^23,29,33,34^, 39–67% against symptomatic infection^23,31^, and 25–60% against hospitalization^26,29^ or hospitalization or death^31^ (Fig. 2). VE of one dose plus prior infection was higher than the VE of one dose without prior infection^21,29,30,32^, and was comparable to two doses plus prior infection in 7 of 9 estimates across multiple endpoints (Table 2, Supplementary Table 4)^26,29,31,33^.

### VE by time since dose

Time since receipt of a single mRNA dose was inconsistently reported in eligible studies. Five studies provided no information on time since a single dose in their analyses^23,24,30,33,34^, while eight studies noted only that VE was measured at least 7 or 14 days after receipt of the dose (Fig. 2)^18,22,25–27,31,32,35^. Five studies reported the range (i.e., minimum and maximum) or median time since dose for single dose VE estimates^19–21,28,29^; follow-up ranged from a median of 14 days^20^, to up to a range of 14 months^29^. Only 4 of 18 studies reported single dose VE estimates stratified by time since dose^19,21,23,29^.

### VE in children and adolescents

During both pre-Omicron and Omicron periods, one dose of original mRNA vaccine plus prior infection provided effective protection against infection in children aged 5–11 years (5 of 6 estimates)^23,28,33^, and against infection (4 of 5 estimates)^23,33^ and symptomatic infection (4 of 5 estimates)^21,23^ in adolescents aged 12–16 or 12–17 years (Fig. 2).

### VE in the elderly

Two studies focused on elderly populations^20,32^. A study of adults aged ≥60 years during Omicron reported strong protection against hospitalization for a single dose plus prior infection during periods of BA.1, BA.2, and BA.4/5 variant predominance^32^. A pre-Omicron study of nursing home residents reported high VE against infection when compared to unvaccinated individuals without prior infection, but found no evidence of added protection with vaccination when compared to unvaccinated residents with prior infection^20^.

### Heterogeneity in studies

Studies had significant heterogeneity in study populations and endpoints. After grouping estimates from the 18 included studies by the five pre-defined groups for synthesis, the largest group size achieved was two studies, for two groups (Supplementary Fig. 1). The high degree of heterogeneity prevented the calculation of median VE by group, meta-analysis, and synthesis of the impact of other factors on VE, including time since dose, order of infection and vaccination, and SARS-CoV-2 variant.

### Risk of bias in studies

In the Newcastle-Ottawa Quality Assessment, 15 studies scored eight or nine stars, and three studies scored seven stars (Supplementary Tables 6–7). No studies scored below the seven-star threshold for exclusion. The two case-control studies that scored seven stars had increased risk of bias primarily due to self-reported ascertainment of vaccination status^22,28^. While four cohort studies were conducted in high-risk populations including health system workers and nursing home residents^18–20,34^, the single cohort study that scored seven stars lacked details describing ascertainment of vaccination status in a health system worker population^34^. Five studies reported ≥1 VE estimate with 95% CIs spanning ≥50 percentage points (Supplementary Tables 3–5)^20,23,27,29,31^, which suggests low certainty of evidence for these estimates.

## Discussion

We found that among individuals with a history of prior SARS-CoV-2 infection, a single COVID-19 mRNA vaccine dose provided significant added protection against infection, symptomatic infection, hospitalization, and hospitalization or death on top of the protection acquired from natural infection alone during both pre-Omicron and Omicron predominance periods^23–26,29,31,33,34^. Effectiveness for one dose plus prior SARS-CoV-2 infection was comparable to protection following two doses plus prior infection, and was higher than protection following two doses without prior infection. While other reviews have summarized COVID-19 VE of multiple doses in individuals with a history of prior SARS-CoV-2 infection^36–38^, to our knowledge this is the first systematic review to summarize the effectiveness of a single dose of mRNA vaccine.

Single dose VE for the original vaccine plus prior infection tended to be higher during the pre-Omicron period than the Omicron period against infection and symptomatic infection. This observation is consistent with VE estimates for two and three doses of original vaccine and may be explained by a better match of vaccine composition to circulating variants or less waning of immunity in pre-Omicron periods^39–41^. Further research is needed to better understand the protection provided by a single dose, particularly for updated formulations of COVID-19 mRNA vaccine, in the current (2023 and onwards) setting of high population-level immunity to SARS-CoV-2 globally.

We did not find any studies that estimated single dose VE among children aged <5 years with history of prior SARS-CoV-2 infection. Considering only 9% of children aged <2 years and 11% of children aged 2–4 years have received ≥1 COVID-19 vaccine dose in the United States as of May 2023^42^, this finding was not unexpected. For older children and adolescents, the available evidence supports the effectiveness of one dose plus prior infection against infection and symptomatic infection^21,23,28,33^, although no data were available for hospitalization or severe outcomes.

Our findings are consistent with immunogenicity studies showing that a single mRNA vaccine dose elicits both strong cellular and humoral responses in those with prior SARS-CoV-2 infection^43–46^. In addition, our results suggesting that one dose plus prior infection provides a higher level of protection than two doses without prior infection are consistent with laboratory studies demonstrating higher concentrations of serum RBD-specific and spike protein-specific IgG antibodies following a single mRNA dose in previously infected individuals compared to those without prior infection who have received two mRNA doses^47–50^. The consistency of evidence on immunogenicity and real-world effectiveness following a single dose supports the continued importance of vaccination against COVID-19 in individuals who have been previously infected, and furthermore, suggests that a single mRNA vaccine dose is likely to provide effective protection against COVID-19 in populations with high pre-existing immunity.

This systematic review is subject to at least eight limitations. First, this review identified only original wild-type mRNA vaccine estimates. To our knowledge, no studies have been published on single dose VE of the bivalent or monovalent XBB.1.5-adapted formulations among those with prior SARS-CoV-2 infection. We anticipate bivalent VE may be similar or higher than VE for original vaccines based on evidence that bivalent vaccine boosters increased protection more than original boosters during Omicron BA.4/5, BQ.1, and XBB predominance^51,52^. Second, our pre-defined groups for aggregating studies to account for heterogeneity may have been too narrow, resulting in few estimates per group that prevented calculation of summary estimates or meta-analysis that tend to provide clearer, more generalizable findings. Nonetheless, general trends in effectiveness for a single vaccine dose plus prior infection remained evident. Third, some studies had small sample size in analyses of single dose VE. Overall, VE estimates were consistent across studies despite heterogeneity in sample size. Fourth, because our review focused on patients with prior SARS-CoV-2 infection, our findings may be subject to selection bias, specifically collider bias, if the requirement for previous infection created an artificial association between vaccination status and the risk of reinfection^53^. Fifth, relevant studies could have been missed in this review if publications used non-standard terminology for the topics of prior infection or vaccine effectiveness. The search strategy was generated in consultation with a reference librarian and reference lists of 11 related systematic reviews and meta-analyses were reviewed without identification of missed studies. Sixth, though only 18 studies were identified for inclusion, this likely reflects the limited available evidence on single dose VE among persons with prior infection, though these findings could be subject to reporting bias. Seventh, few studies reported information on time since vaccine receipt for their VE estimates. Although four studies reported single dose VE estimates stratified by time since dose^19,21,23,29^, the intervals differed across studies and prevented assessment of VE over time. Further research is necessary on the durability of protection for single dose vaccination following infection. Eighth, no studies were identified reporting one dose VE in immunocompromised populations, children aged <5 years, or from certain geographic regions of the world, indicating important data gaps.

Findings from this systematic review support recent policy recommendations and decisions for receipt of one COVID-19 vaccine dose regardless of vaccination history for immunocompetent individuals ≥5 years of age for the 2023–2024 and 2024–2025 respiratory virus seasons. Of note, we did observe that a single dose in the absence of prior SARS-CoV-2 infection provided protection during pre-Omicron and Omicron periods, although this protection tended to be lower than that provided by hybrid immunity of one dose and prior infection. This review also indicates, however, that there is a lack of published, peer-reviewed evidence describing the protection conferred by a single dose among those with prior SARS-CoV-2 infection for certain populations, including young children and immunocompromised persons. Due to the increased risk of severe disease in these populations^54,55^ and the gap in evidence, additional doses continue to be warranted in these groups in alignment with current recommendations for additional doses for certain patient populations^8,9^. Because of waning protection following infection, vaccination, or both, and the continuing emergence of antigenically distinct variants^5,6^, protection against SARS-CoV-2 infection and severe COVID-19 outcomes will continue to fluctuate. To maintain optimal protection, COVID-19 vaccination should be given annually before the viral respiratory season to the general population^56,57^, and additional doses may be needed for certain populations. Thus, the effectiveness of COVID-19 vaccines should be continually monitored to inform future vaccine recommendations in real time.

In summary, a single dose of COVID-19 vaccine improves protection against a range of COVID-19 outcomes and provides similar protection to that conferred by a two-dose series for immunocompetent individuals aged ≥5 years in the current setting of high pre-existing SARS-CoV-2 immunity. These findings support current recommendations for one dose of COVID-19 vaccines to be given in advance of the viral respiratory season, regardless of prior history of SARS-CoV-2 infection or COVID-19 vaccination, with considerations for additional doses for certain populations including young children, older adults, and the immunocompromised.

## Supplementary information

SUPPLEMENTARY MATERIALS

REPORTING SUMMARY

## Data Availability

All data generated or analyzed during this study are included in this published article and its supplementary information files. The Tables, Figures, and Supplementary Information summarize data exactly as reported by the original study authors in the 18 included studies.
