# Supplementary material for: Effectiveness of a single COVID-19 mRNA vaccine dose in individuals with prior SARS-CoV-2 infection: a systematic review
Source: Commun Med (Lond). 2025 May 3;5:151. doi: 10.1038/s43856-025-00882-y (PMC12049417; doi:10.1038/s43856-025-00882-y)
Supplement: Supplementary file 1 — SUPPLEMENTARY MATERIALS [file 43856_2025_882_MOESM1_ESM.pdf]

SUPPLEMENTARY MATERIALS

Effectiveness of a Single COVID-19 mRNA Vaccine Dose in Individuals with Prior SARS-CoV-2 Infection: A Systematic Review.

Hannah R Volkman; Jennifer L Nguyen; Mustapha M Mustapha; Jingyan Yang; Luis Jodar; John M McLaughlin

Supplementary Figure 1. Harvest plot of number of studies within each group defined by variant period, outcome, age group, referent group, and sub-variant period

Supplementary Table 1. Search strategy for systematic review and results of search on October 4, 2023

Supplementary Table 2. Characteristics of 18 studies reporting single dose COVID-19 mRNA vaccine effectiveness among immunocompetent individuals with prior infection

Supplementary Tables 3–5. Summary of all vaccine effectiveness estimates extracted from included studies

Supplementary Tables 6–7. Bias assessment using the Newcastle-Ottawa Quality Assessment Scale

Supplementary References

Supplementary Figure 1. Harvest plot of number of studies within each group defined by referent group, variant period, outcome, age group, and predominant sub-variant

Age group	Pre-Omicron				Omicron			
	Infection	Symptomatic infection	Hospitalization	Hospitalization or death	Infection	Symptomatic infection	Hospitalization	Hospitalization or death
**0–4y**	0	0	0	0	0	0	0	0
**5–11y**	1 1PI, 0V	0	0	0	1 0PI, 0V - BA.1,2,4,5<br>1 1PI, 0V - BA.1,2<br>2 1PI, 0V - BA.4,5<br>1 1PI, 0V - XBB	0	0	0
**12–16/17y**	1 1PI, 0V	1 0PI, 0V<br>1 1PI, 0V	0	0	1 1PI, 0V - BA.1,2<br>2 1PI, 0V - BA.4,5<br>1 1PI, 0V - XBB	1 0PI, 0V - BA.1,2<br>1 1PI, 0V - BA.1,2<br>1 1PI, 0V - BA.4,5	0	0
**≥12y**	1 1PI, 0V	0	0	0	1 0PI, 0V - BA.1<br>1 1PI, 0V - BA.1<br>1 0PI, 2V - BA.1,2	0	1 0PI, 0V - BA.1<br>1 1PI, 0V - BA.1<br>1 0PI, 2V - BA.1,2	0
**≥16y**	1 1PI, 2V	0	0	0	0	0	0	0
**≥18y**	1 0PI, 0V	1 0PI, 0V<br>1 1PI, 0V	1 1PI, 0V	1 1PI, 0V	1 0PI, 0V - BA.2	1 0PI, 0V - BA.1<br>1 0PI, 0V - BA.2<br>1 1PI, 0V - BA.1	1 1PI, 0V - BA.1	1 0PI, 0V - BA.1<br>1 1PI, 0V - BA.1
**18–64y**	1 0PI, 0V	0	0	0	0	0	0	0
**≥60y**	0	0	0	0	0	0	1 0PI, 0V - BA.1<br>1 0PI, 0V - BA.2<br>1 0PI, 0V - BA.4,5	0
**Working age**	0	0	0	0	1 1PI, 0V - BA.1<br>1 1PI, 1V - BA.1	0	0	0
**Nursing home residents**	1 0PI, 0V<br>1 1PI, 0V	0	0	0	0	0	0	0

Referent group color key:

0PI, 0V: Immunologically naïve (No prior infection, unvaccinated)

1PI, 0V: Prior infection, unvaccinated

Other type of referent group, noted

Supplementary Table 1. Search strategy for systematic review and results of search on October 4, 2023

Search number	Description	Content	Results
1	COVID-19 terms	exp *coronavirus disease 2019/ or exp *SARS-CoV-2 Omicron/	478,357
2		("2019 novel coronavirus" or 2019-nCoV or "coronavirus disease 2" or "coronavirus disease 2019" or coronavirus disease-19 or "coronavirus infection 2019" or COVID or COVID-19 or COVID19 or "nCoV 2019" or "SARS coronavirus 2" or SARS-CoV-2 or SARS-CoV2 or SARSCoV2 or "severe acute respiratory syndrome 2" or "severe acute respiratory syndrome coronavirus 2 infection" or "severe acute respiratory syndrome coronavirus 2019 infection" or "severe acute respiratory syndrome CoV-2 infection" or Wuhan coronavirus).ti,kw,kf.	703,403
3		exp *COVID-19/	477,046
4	COVID-19 vaccine terms	exp SARS-CoV-2 vaccine/	63,781
5		((SARS-CoV-2 or 2019-nCoV or "coronavirus disease 2019" or "COVID 19" or COVID-19 or COVID19 or HCoV-19 or "coronavirus 2019" or nCoV-2019 or "novel 2019 coronavirus" or "novel coronavirus 2019" or "SARS Coronavirus 2" or SARS2 or "severe acute respiratory syndrome 2" or "severe acute respiratory syndrome coronavirus 2" or Wuhan coronavirus) adj2 (vaccin$ or innoculat$)).ti,ab.	72,600
6		exp COVID-19 Vaccines/	63,781
7		(mRNA or messenger RNA).mp.	1,506,172
8		exp mRNA Vaccines/	21,954
9		exp elasomeran/	7,177
10		(Elasomeran or mRNA-1273 or mRNA1273 or RNA-1273 or RNA1273 or spikevax).ti,ab.	4,475
11		exp tozinameran/	14,275
12		(Tozinameran or ((biontech or Pfizer) adj3 vaccine) or bnt 162b2 or bnt162b2 or comirnaty).ti,ab.	13431
13	Combination of above terms	1 or 2 or 3	739,637
14		4 or 5 or 6	90,094
15		7 or 8	1,515,027
16		14 and 15	27,925
17		9 or 10 or 11 or 12 or 16 or *SARS-CoV-2 vaccine/dt	36,332
18		13 and 17	34,256
19	Prior infection terms	exp reinfection/ or "hybrid immunity"/	47,138
20		(reinfect$ or re-infect$ or ((prior or previous$ or secondary) adj3 infect$)).ti,ab.	129,429
21	Combination of above terms	19 or 20	165,318
22		18 and 21	2,266
23	De-duplication	remove duplicates from 22	1,661

Supplementary Table 2. Characteristics of 18 studies reporting single dose COVID-19 mRNA vaccine effectiveness among immunocompetent individuals with prior infection

Author, Journal, Year	Country	Vaccine	Study design	Predominant variant	Study period	Age group	Total sample size
Carazo et al., JAMA Netw Open, 2022[29]	Canada	BNT162b2 and mRNA-1273	Test negative case-control	Omicron BA.1	Dec 26, 2021 to Mar 12, 2022	≥12 y	696,439
Carazo et al., Lancet Healthy Longev, 2023[32]	Canada	BNT162b2 and mRNA-1273	Test negative case-control	Omicron BA.1	Dec 26, 2021 to Mar 12, 2022	≥60y	51,675
				Omicron BA.2	Apr 3, 2022 to Jun 11, 2022	≥60y	43,599
				Omicron BA.4 and BA.5	Jul 3, 2022 to Nov 5, 2022	≥60y	79,545
Carazo et al., Lancet Infect Dis, 2023[30]	Canada	BNT162b2 and mRNA-1273	Test negative case-control	Omicron BA.2	Mar 27, 2022 to Jun 4, 2022	≥18 y	111,239
Cerqueira-Silva et al., Lancet Infect Dis, 2022[25]	Brazil	BNT162b2	Test negative case-control	Wild-type, Delta	Jan 18, 2021 to Nov 11, 2021	≥18 y	90,992
Cerqueira-Silva et al., Lancet Infect Dis, 2022[31]	Brazil	BNT162b2	Test negative case-control	Omicron BA.1	Jan 1, 2022 to Mar 22, 2022	≥18 y	899,050
Gazit et al., Lancet Microbe, 2023[23]	Israel	BNT162b2	Retrospective cohort - target trial emulation	Delta	Jun 2, 2021 to Dec 2021	5–11 y	120,721
						12–16 y	43,091
				Omicron BA.1 and BA.2	Jan 2022 to Mar 2022	5–11 y	120,721
						12–16 y	43,091
				Omicron BA.4 and BA.5	Apr 2022 to Jul 31, 2022	5–11 y	120,721
						12–16 y	43,091
Grant et al., Lancet Reg Health Eur, 2022[22]	France	BNT162b2 and mRNA-1273	Case control	Delta	May 23, 2021 to Aug 13, 2021	≥18 y	18,194
Hall et al., N Engl J Med, 2022[19]	United Kingdom	BNT162b2	Prospective cohort	Wild-type, Delta	Dec 7, 2020 to Sep 21, 2021	≥18 y	35,768
Hammerman et al., N Engl J Med, 2022[27]	Israel	BNT162b2	Retrospective cohort	Alpha, Delta	Mar 1, 2021 to Nov 26, 2021	≥16 y	149,032
Hatfield et al., Clin Infect Dis, 2022[20]	United States	BNT162b2 and mRNA-1273	Retrospective cohort	Delta	Dec 14, 2020 to Nov 9, 2021	Nursing home residents	4,315
Huang et al., NPJ Vaccines, 2022[35]	China (Hong Kong)	BNT162b2	Retrospective cohort	Omicron BA.1 and BA.2	Jan 1, 2022 to Mar 31, 2022	≥12 y	1,561,794
Khan et al., JAMA Netw Open, 2022[28]	United States	BNT162b2	Test negative case-control	Omicron BA.1, BA.2, BA.4, and BA.5	Jan 16, 2022 to Sep 30, 2022	5–11 y	160,002
Lewis et al., JAMA Netw Open, 2022[24]	United States	BNT162b2 and mRNA-1273	Retrospective cohort	Wild-type, Alpha, Delta	Dec 2020 to Dec 9, 2021	≥12 y	94,516
Peebles, J Infect Dis, 2023[18]	United States	BNT162b2	Retrospective cohort	Wild-type, Iota, Alpha, Delta	Jan 21, 2021 to Jun 5, 2021	18–64 y	8,602
Plumb et al., MMWR Morb Mortal Wkly Rpt, 2022[26]	United States	BNT162b2 and mRNA-1273	Test negative case-control	Delta	Jun 20, 2021 to Dec 18, 2021	≥18 y	4,385
				Omicron BA.1	Dec 19, 2021 to Feb 24, 2022		6,898
Powell et al., Lancet Infect Dis, 2023[21]	United Kingdom	BNT162b2	Test negative case-control	Delta	Aug 9, 2021 to Nov 2021	12–17 y	851,223
				Omicron BA.1 and BA.2	Nov 2021 to Mar 31, 2022		437,880
Shrestha et al., Clin Infect Dis, 2022[34]	United States	BNT162b2 and mRNA-1273	Retrospective cohort	Omicron BA.1	Nov 26, 2021 to Jan 28, 2022	Not described; working age	39,766
Yung et al., Lancet Child Adolesc Health, 2023[33]	Singapore	BNT162b2	Retrospective cohort	Omicron BA.4 and BA.5	Jun 1, 2022 to Sep 30, 2022	5–11 y	79,332
						12–17 y	55,865
				Omicron XBB	Oct 18, 2022 to Dec 15,2022	5–11 y	97,235
						12–17 y	67,469

Supplementary Table 3. Summary of VE estimates where the referent group is immunologically naïve (unvaccinated and no history of prior SARS-CoV-2 infection)

Author, Journal, Year	Age group	COVID-19 Outcome	Sub-variant	VE: 0 doses, prior infection	VE: 1 dose, no prior infection	VE: 1 dose, prior infection	VE: 2 doses, no prior infection	VE: 2 doses, prior infection	VE: 3 doses, no prior infection	VE: 3 doses, prior infection
Pre-Omicron										
Peebles et al., *J Infect Dis*, 2023[18]	18–64 y	Infection	–	64.2% (41.5 to 78.0)	73.8% (49.8 to 86.4)	79.6% (61.8 to 89.1)	83.9% (74.4 to 89.9)	85.0% (76.9 to 90.2)	–	–
Hall et al., *N Engl J Med*, 2022[19]	≥18 y	Infection	–	86% (81 to 89)	59% (42 to 71)	92% (86 to 95)	85% (72 to 92)	84% (67 to 92)	–	–
Hatfield et al., *Clin Infect Dis*, 2022[20]	Nursing home residents	Infection	–	58% (26 to 76)	34% (10 to 52)	78% (56 to 89)	58% (44 to 69)	78% (67 to 85)	–	–
Powell et al., *Lancet Infect Dis*, 2023[21]	12–17 y	Symptomatic infection	–	*Previous Alpha:* 86.1% (85.4 to 86.8) *Previous Delta:* 92.3% (90 to 97)	59.4% (58.8 to 60.0)	98.1% (97.6 to 98.6)	91.8% (91.2 to 92.3)	98.8% (96.7 to 99.5)	96.0% (92.2 to 97.9)	–
Grant et al., *Lancet Reg Health Eur*, 2022[22]	≥18 y	Symptomatic infection	–	*Infection 2-6 mo. ago:* 95% (90 to 97) *Infection >6 mo.:* 74% (58 to 84)	22% (10 to 32)	85% (78 to 90)	67% (63 to 71)	96% (87 to 99)	–	–
Omicron										
Khan et al., *JAMA Netw Open*, 2022[28]	5–11 y	Infection	BA.1/2/4/5	–	12% (4 to 19)	32% (12 to 48)	19% (16 to 22)	36% (28 to 44)	51% (44 to 57)	70% (60 to 78)
Carazo et al., *JAMA Netw Open*, 2022[29]	≥12 y	Infection	BA.1	44% (38 to 48)	20% (16 to 24)	65% (63 to 67)	42% (41 to 44)	68% (67 to 70)	73% (72 to 73)	83% (81 to 84)
Carazo et al., *Lancet Infect Dis*, 2023[30]	≥18 y	Infection	BA.2	72% (65 to 78)	9% (-19 to 31)	89% (78 to 95)	35% (27 to 42)	96% (95 to 96)	46% (40 to 52)	96% (95 to 97)
Powell et al., *Lancet Infect Dis*, 2023[21]	12–17 y	Symptomatic infection	BA.1/2	*Previous wild-type:* 32.7% (27.7 to 37.4) *Previous Omicron:* 59.3% (46.7 to 69.0)	18.8% (17.2 to 20.3)	85.3% (83.7 to 86.8)	64.5% (63.6 to 65.4)	84.7% (82.6 to 86.5)	62.9% (60.5 to 65.1)	79.8% (70.4 to 86.3)
Carazo et al., *Lancet Infect Dis*, 2023[30]	≥18 y	Symptomatic infection	BA.2	86% (79 to 91)	43% (11 to 63)	97% (89 to 99)	61% (52 to 69)	98% (97 to 98)	70% (62 to 75)	98% (98 to 99)
Cerqueira-Silva et al., *Lancet Infect Dis*, 2022[31]	≥18 y	Symptomatic infection	BA.1	31.7% (30.0 to 33.4)	–	57.1% (55.8 to 58.3)	–	66.5% (65.5 to 67.5)	–	70.0% (68.4 to 71.6)
Carazo et al., *JAMA Netw Open*, 2022[29]	≥12 y	Hospitalization	BA.1	81% (66 to 89)	52% (42 to 61)	86% (77 to 91)	76% (74 to 78)	94% (91 to 96)	91% (91 to 92)	97% (94 to 99)
Carazo et al., *Lancet Healthy Longev*, 2023[32]	≥60 y	Hospitalization	BA.1	93% (80 to 97)	62% (50 to 71)	94% (85 to 98)	78% (75 to 80)	97% (96 to 99)	93% (92 to 93)	98% (96 to 99)
Carazo et al., *Lancet Healthy Longev*, 2023[32]	≥60 y	Hospitalization	BA.2	88% (50 to 97)	43% (9 to 64)	80% (42 to 93)	60% (50 to 67)	92% (86 to 96)	75% (71 to 79)	94% (90 to 97)
Carazo et al., *Lancet Healthy Longev*, 2023[32]	≥60 y	Hospitalization	BA.4/5	69% (30 to 85)	32% (0 to 53)	94% (60 to 99)	40% (30 to 49)	92% (80 to 97)	59% (53 to 64)	88% (81 to 92)
Cerqueira-Silva et al., *Lancet Infect Dis*, 2022[31]	≥18 y	Hospitalization or death	BA.1	81.9% (76.2 to 86.3)	–	88.9% (82.2 to 93.1)	–	90.9% (84.0 to 94.8)	–	95.7% (90.6 to 98.0)

Red VE estimates have a 95% CI spanning ≥50 percentage points

Supplementary Table 4. Summary of VE estimates where the referent group is unvaccinated and has history of prior SARS-CoV-2 infection

Author, Journal, Year	Age group	COVID-19 Outcome	Sub-variant	VE: 1 dose, prior infection	VE: 2 doses, prior infection	VE: 3 doses, prior infection
Pre-Omicron						
Gazit et al., *Lancet Microbe*, 2023[23]	5–11 y	Infection	–	64.0% (2.5 to 86.8)	–	–
Gazit et al., *Lancet Microbe*, 2023[23]	12–16 y	Infection	–	78.2% (71.6 to 83.2)	–	–
Lewis et al., *JAMA Netw Open*, 2022[24]	≥12 y	Infection	–	52% (37 to 64)	64% (58 to 69)	–
Hatfield et al., *Clin Infect Dis*, 2022[20]	Nursing home residents	Infection	–	11% (-98 to 60)	11% (-56 to 49)	–
Gazit et al., *Lancet Microbe*, 2023[23]	12–16 y	Symptomatic infection	–	76.6% (64.0 to 84.8)	–	–
Cerqueira-Silva et al., *Lancet Infect Dis*, 2022[25]	≥18 y	Symptomatic infection	–	45.0% (39.7 to 49.9)	64.8% (54.9 to 72.4)	–
Plumb et al., *MMWR*, 2022[26]	≥18 y	Hospitalization	–	58.8% (41.3 to 71.1)	47.5% (38.8 to 54.9)	57.8% (32.1 to 73.8)
Cerqueira-Silva et al., *Lancet Infect Dis*, 2022[25]	≥18 y	Hospitalization or death	–	61.8% (40.8 to 75.3)	89.7% (54.3 to 97.7)	–
Omicron						
Gazit et al., *Lancet Microbe*, 2023[23]	5–11 y	Infection	BA.1/2	70.5% (67.0 to 73.0)	–	–
Gazit et al., *Lancet Microbe*, 2023[23]	5–11 y	Infection	BA.4/5	12.1% (-5.7 to 26.9)	–	–
Yung et al., *Lancet Child Adolesc Health*, 2023[33]	5–11 y	Infection	BA.4/5	44.0% (32.2 to 53.7)	74.0% (67.7 to 79.1)	–
Yung et al., *Lancet Child Adolesc Health*, 2023[33]	5–11 y	Infection	XBB	59.1% (35.7 to 73.9)	62.8% (42.3 to 76.0)	–
Gazit et al., *Lancet Microbe*, 2023[23]	12–16 y	Infection	BA.1/2	53.7% (50.1 to 57.0)	–	–
Gazit et al., *Lancet Microbe*, 2023[23]	12–16 y	Infection	BA.4/5	8.1% (-18.3 to 28.6)	–	–
Yung et al., *Lancet Child Adolesc Health*, 2023[33]	12–17 y	Infection	BA.4/5	70.4% (53.3 to 81.2)	84.9% (77.0 to 90.1)	85.7% (80.2 to 89.6)
Yung et al., *Lancet Child Adolesc Health*, 2023[33]	12–17 y	Infection	XBB	54.5% (22.1 to 73.4)	57.9% (33.6 to 73.3)	47.9% (20.2 to 66.1)
Carazo et al., *JAMA Netw Open*, 2022[29]	≥12 y	Infection	BA.1	40% (33 to 46)	45% (40 to 50)	70% (67 to 73)
Shrestha et al., *Clin Infect Dis*, 2022[34]	Not described, working age	Infection	BA.1	59.0% (46.8 to 68.3)	–	–
Gazit et al., *Lancet Microbe*, 2023[23]	12–16 y	Symptomatic infection	BA.1/2	67.4% (57.1 to 75.2)	–	–
Gazit et al., *Lancet Microbe*, 2023[23]	12–16 y	Symptomatic infection	BA.4/5	62.1% (-40.4 to 90.0)	–	–
Cerqueira-Silva et al., *Lancet Infect Dis*, 2022[31]	≥18 y	Symptomatic infection	BA.1	39.2% (36.7 to 41.6)	54.1% (52.1 to 55.9)	58.1% (55.3 to 60.6)
Carazo et al., *JAMA Netw Open*, 2022[29]	≥12 y	Hospitalization	BA.1	25% (-56 to 64)	70% (39 to 85)	85% (62 to 94)
Plumb et al., *MMWR*, 2022[26]	≥18 y	Hospitalization	BA.1	33.0% (15.0 to 47.2)	34.6% (25.5 to 42.5)	67.6% (61.4 to 72.8)
Cerqueira-Silva et al., *Lancet Infect Dis*, 2022[31]	≥18 y	Hospitalization or death	BA.1	60.0% (15.4 to 81.1)	53.6% (-6.4 to 79.8)	85.2% (55.7 to 95.1)

Red VE estimates have a 95% CI spanning ≥50 percentage points

Supplementary Table 5. Summary of VE estimates where the referent group is uncommon and defined in the table

Author, Journal, Year	Age group	COVID-19 Outcome	Referent group	Sub-variant	VE: 1 dose, prior infection	VE: 2 doses, prior infection	VE: 3 doses, prior infection
Pre-Omicron							
Hammerman et al., *N Engl J Med,* 2022[27]	≥16 y	Infection	2 doses, prior infection	–	-2% (-56 to 33)	–	–
Omicron							
Huang et al., *NPJ Vaccines*, 2022[35]	≥12 y	Infection	2 doses, no prior infection	BA.1/2	52.5% (45.0 to 59.0)	–	–
Shrestha et al., *Clin Infect Dis,* 2022[34]	Not described, working age	Infection	1 dose, prior infection	BA.1	–	-54% (-97 to -21)	-1% (-32 to 23)
Huang et al., *NPJ Vaccines*, 2022[35]	≥12 y	Hospitalization	2 doses, no prior infection	BA.1/2	60.6% (-4.6 to 85.2)	–	–

Red VE estimates have a 95% CI spanning ≥50 percentage points

Supplementary Table 6. Bias assessment of included studies using the Newcastle-Ottawa Quality Assessment Scale for case-control studies

	Selection				Comparability	Exposure			Score
**Author, Journal, Year**	**1) Is the case definition adequate?**	**2) Representativeness of the cases**	**3) Selection of controls**	**4) Definition of controls**	**1) Comparability of cases and controls on the basis of the design or analysis**	**1) Ascertainment of exposure**	**2) Same method of ascertainment for cases and controls**	**3) Non-response rate**	**Number of stars**
Carazo et al., *Lancet Infect Dis*, 2023[30]	a) yes, with independent validation	a) consecutive or obviously representative series of cases (TND)	a) community controls	a) test negative controls	a) study controls for indication for testing<br>b) analyses stratified by time since last primary infection or vaccine	a) a secure record	a) yes	b) non respondents described	8
Carazo et al., *Lancet Health Longev*, 2023[32]	a) yes, with independent validation	a) consecutive or obviously representative series of cases (TND)	a) community controls	a) test negative controls	a) study controls for epi week<br>b) study controls for additional factors	a) secure record	a) yes	b) non respondents described	8
Carazo et al., *JAMA Netw Open*, 2022[29]	a) yes, with independent validation	a) consecutive or obviously representative series of cases (TND)	a) community controls	a) test negative controls	a) study controls for epi week<br>b) study controls for additional factors	a) a secure record	a) yes	b) non respondents described	8
Cerqueira-Silva et al., *Lancet Infect Dis*, 2022[25]	a) yes, with independent validation	a) consecutive or obviously representative series of cases (TND)	a) community controls	a) test negative controls	a) study controls for days since previous infection<br>b) study controls for additional factors	a) secure record	a) yes	b) non respondents described	8
Cerqueira-Silva et al., *Lancet Infect Dis*, 2022[31]	a) yes, with independent validation	a) consecutive or obviously representative series of cases (TND)	a) community controls	a) test negative controls	a) study controls for calendar week<br>b) study controls for additional factors	a) a secure record	a) yes	b) non respondents described	8
Grant et al., *Lancet Reg Health Eur*, 2022[22]	a) yes, with independent validation	a) consecutive or obviously representative series of cases	a) community controls from a representative sample of the population - some potential for selection bias	a) no history of disease - includes all cases in France so classification as a control necessitates absence of positive result	a) study controls for many SARS-CoV-2 exposure factors<br>b) study controls for additional factors	d) written self-report	b) no	a) same rate for both groups	7
Khan et al., *JAMA Netw Open,* 2022[28]	a) yes, with independent validation	a) consecutive or obviously representative series of cases (TND)	a) community controls	a) test negative controls	a) study controls for previous SARS-CoV-2 infection<br>b) study controls for additional factors	d) written self-report	a) yes	b) non respondents described	7
Plumb et al., *MMWR Morb Mortal Wkly Rpt*, 2022[26]	a) yes, with independent validation	a) consecutive or obviously representative series of cases (TND)	a) community controls	a) test negative controls	a) study controls for days since previous infection<br>b) study controls for additional factors	a) a secure record - unclear if comprehensive	a) yes	c) no designation	8
Powell et al., *Lancet Infect Dis*, 2023[21]	a) yes, with independent validation	a) consecutive or obviously representative series of cases (TND)	a) community controls	a) test negative controls	a) study controls for time period of test<br>b) study controls for additional factors	a) a secure record	a) yes	a) comprehensive, national source for exposure	9

Color key: blue=2 stars; white=1 star; red=0 stars.

Supplementary Table 7. Bias assessment of included studies using the Newcastle-Ottawa Quality Assessment Scale for cohort studies

	Selection				Comparability	Outcome			Score
**Author, Journal, Year**	**1) Representativeness of the exposed cohort**	**2) Selection of the non-exposed cohort**	**3) Ascertainment of exposure**	**4) Demonstration that outcome of interest was not present at start of study**	**1) Comparability of cohorts on the basis of the design or analysis**	**1) Ascertainment of outcome**	**2) Was follow-up long enough for outcomes to occur**	**3) Adequacy of follow up of cohorts**	**Number of stars**
Gazit et al., *Lancet Microbe*, 2023[23]	b) somewhat representative of the average child or adolescent in the community	a) drawn from the same community as the exposed cohort	a) secure record - but if vaccinated outside health system, unclear if it would be documented in record	a) yes, 90-day washout but untested infections might occur leading to misclassification	a) study controls for variant b) study controls for multiple other factors	b) record linkage (SARS-CoV-2 PCR or antigen test, likely done to evaluate symptoms)	a) yes, over 1 year	b) subjects lost to follow up unlikely to introduce bias - retrospective cohort	9
Hall et al., *N Engl J Med*, 2022[19]	c) selected group of users - healthcare workers	a) drawn from the same community as the exposed cohort	a) a secure record	a) yes, tested every 2 weeks and antigen testing monthly, and excluded if an infection occurred after vaccination but before study	a) the study controls for sex and race b) the study stratifies by many relevant factors	b) record linkage (PCR tests done frequently)	a) yes, 9 months	b) subjects lost to follow up unlikely to introduce bias - prospective cohort, follow-up censored	8
Hammerman et al., *N Engl J Med*, 2022[27]	b) somewhat representative of those eligible for vaccination in Israel	a) drawn from the same community as the exposed cohort	a) secure record - but if vaccinated outside system, unclear if it would be documented	a) yes, tests within 100 days were considered part of same episode or illness	a) the study controls for co-existing illnesses b) the study controls for sociodemographic factors	b) record linkage (PCR test)	a) yes, 8 months	b) subjects lost to follow up unlikely to introduce bias - retrospective cohort	9
Hatfield et al., *Clin Infect Dis*, 2022[20]	c) selected group of users - nursing home residents	a) drawn from the same community as the exposed cohort	a) secure record	a) yes, 90-day washout but untested infections might occur leading to misclassification	a) study controls for comorbid conditions b) the study controls for multiple other factors	b) record linkage (PCR test)	a) yes, 11 months	b) subjects lost to follow up unlikely to introduce bias - retrospective cohort	8
Huang et al., *NPJ Vaccines*, 2022[35]	a) truly representative of the Hong Kong population	a) drawn from the same community as the exposed cohort	a) secure record	a) yes, excluded those who received one dose and were infected before Omicron and those with two doses and prior infection	a) study controls for time since last SARS-CoV-2 infection b) study controls for multiple other covariates	b) record linkage (PCR test)	a) yes, 3 months	b) subjects lost to follow up unlikely to introduce bias - retrospective cohort	9
Lewis et al., *JAMA Netw Open*, 2022[24]	a) truly representative of Rhode Island residents	a) drawn from the same community as the exposed cohort	a) a secure record	a) yes, 90-day washout but untested infections might occur leading to misclassification	a) the study controls for time of entry into the cohort b) the study controls for multiple other covariates	b) record linkage (statewide surveillance data)	a) yes, 3 months	b) subjects lost to follow up unlikely to introduce bias - retrospective cohort	9
Peebles et al., *J Infect Dis*, 2023[18]	c) selected group of users - long-term care employees	a) drawn from the same community as the exposed cohort	a) secured record using probabilistic matching - risk of misclassification exists	a) yes, tested every 10 days or less, and those infected in the last 90 days excluded	a) study controls for calendar week b) study controls for multiple other covariates	b) record linkage (PCR tests done frequently)	a) yes, 6 months	b) subjects lost to follow up unlikely to introduce bias - retrospective cohort	8
Shrestha et al., *Clin Infect Dis*, 2022[34]	c) selected group of users - clinic employees	a) drawn from the same community as the exposed cohort	d) no description	a) yes, tests within 90 days were considered part of same episode or illness	a) study controls for time since proximate SARS-CoV-2 exposure b) study controls for multiple other factors	b) record linkage (SARS-CoV-2 tests done to evaluate symptoms, or work exposure)	a) yes, approx. 63 days - end date not described	b) subjects lost to follow up unlikely to introduce bias - retrospective cohort	7
Yung et al., *Lancet Child Adolesc Health*, 2023[33]	a) truly representative of children and adolescents in Singapore	a) drawn from the same community as the exposed cohort	a) a secure record	a) yes, 90-day washout but untested infections might occur leading to misclassification	a) study controls for calendar week b) study controls for multiple other factors	b) record linkage (PCR test)	a) yes, 6 months	b) subjects lost to follow up unlikely to introduce bias - retrospective cohort	9

Color key: blue=2 stars; white=1 star; red=0 stars.

Supplementary References

1 Jones, J. *et al.* Estimates of SARS-CoV-2 Seroprevalence and Incidence of Primary SARS-CoV-2 Infections Among Blood Donors, by COVID-19 Vaccination Status — United States, April 2021–September 2022. *MMWR Morb Mortal Wkly Rep* **72**, 601-605 (2023). https://doi.org:10.15585/mmwr.mm7222a3

2 Skowronski, D. M. *et al.* Serial cross-sectional estimation of vaccine-and infection-induced SARS-CoV-2 seroprevalence in British Columbia, Canada. *CMAJ* **194**, E1599-E1609 (2022). https://doi.org:10.1503/cmaj.221335

3 United Kingdom Office for National Statistics. *Coronavirus (COVID-19) latest insights: Antibodies*, <https://www.ons.gov.uk/peoplepopulationandcommunity/healthandsocialcare/conditionsanddiseases/articles/coronaviruscovid19latestinsights/antibodies> (2023).

4 Kislaya, I. *et al.* Seroprevalence of Specific SARS-CoV-2 Antibodies during Omicron BA.5 Wave, Portugal, April-June 2022. *Emerg Infect Dis* **29**, 590-594 (2023). https://doi.org:10.3201/eid2903.221546

5 van der Straten, K. *et al.* Antigenic cartography using sera from sequence-confirmed SARS-CoV-2 variants of concern infections reveals antigenic divergence of Omicron. *Immunity* **55**, 1725-1731 (2022). https://doi.org:10.1016/j.immuni.2022.07.018

6 Goldberg, Y. *et al.* Protection and Waning of Natural and Hybrid Immunity to SARS-CoV-2. *N Engl J Med* **386**, 2201-2212 (2022). https://doi.org:10.1056/nejmoa2118946

7 United States Food and Drug Administration. *Coronavirus (COVID-19) Update: FDA Authorizes Changes to Simplify Use of Bivalent mRNA COVID-19 Vaccines*, <https://www.fda.gov/news-events/press-announcements/coronavirus-covid-19-update-fda-authorizes-changes-simplify-use-bivalent-mrna-covid-19-vaccines> (2023).

8 United States Food and Drug Administration. *FDA Takes Action on Updated mRNA COVID-19 Vaccines to Better Protect Against Currently Circulating Variants*, <https://www.fda.gov/news-events/press-announcements/fda-takes-action-updated-mrna-covid-19-vaccines-better-protect-against-currently-circulating> (2023).

9 European Medicines Agency. *Comirnaty: EMA recommends approval of adapted COVID-19 vaccine targeting Omicron XBB.1.5*, <https://www.ema.europa.eu/en/news/comirnaty-ema-recommends-approval-adapted-covid-19-vaccine-targeting-omicron-xbb15> (2023).

10 World Health Organization. *Highlights from the Meeting of the Strategic Advisory Group of Experts (SAGE) on Immunization 25-29 September 2023*, <https://cdn.who.int/media/docs/default-source/immunization/sage/2023/september/sage_sept2023_meeting_highlights.pdf?sfvrsn=5ac08c01_4> (2023).

11 Page, M. J. *et al.* PRISMA 2020 explanation and elaboration: updated guidance and exemplars for reporting systematic reviews. *BMJ* **372** (2021). https://doi.org:10.1136/bmj.n160

12 Campbell, M. *et al.* Synthesis without meta-analysis (SWiM) in systematic reviews: reporting guideline. *BMJ* **368** (2020). https://doi.org:10.1136/bmj.l6890

13 Hodcroft, E. *CoVariants - Overview of Variants in Countries*, <https://covariants.org/per-country> (2023).

14 Lievens, M. *et al.* Statistical methodology for the evaluation of vaccine efficacy in a phase III multi-centre trial of the RTS,S/AS01 malaria vaccine in African children. *Malar J* **10**, 222 (2011). https://doi.org:10.1186/1475-2875-10-222

15 Wells, G. *et al. The Newcastle-Ottawa Scale (NOS) for assessing the quality of nonrandomised studies in meta-analyses*, <https://www.ohri.ca/programs/clinical_epidemiology/oxford.asp> (2000).

16 Rane, M. S. *et al.* Effectiveness of Covid-19 vaccines against symptomatic and asymptomatic SARS-CoV-2 infections in an urgent care setting. *Vaccine* **41**, 989-998 (2023). https://doi.org:10.1016/j.vaccine.2022.12.039
17 Suarez Castillo, M., Khaoua, H. & Courtejoie, N. Vaccine-induced and naturally-acquired protection against Omicron and Delta symptomatic infection and severe COVID-19 outcomes, France, December 2021 to January 2022. *Euro Surveill* **27**, 1-7 (2022). https://doi.org:10.2807/1560-7917.es.2022.27.16.2200250
18 Peebles, K. *et al.* Pfizer-BioNTech Coronavirus Disease 2019 Vaccine Effectiveness Against Severe Acute Respiratory Syndrome Coronavirus 2 Infection Among Long-term Care Facility Staff With and Without Prior Infection in New York City, January-June 2021. *J Infect Dis* **227**, 533-542 (2023). https://doi.org:10.1093/infdis/jiac448
19 Hall, V. *et al.* Protection against SARS-CoV-2 after Covid-19 Vaccination and Previous Infection. *N Engl J Med* **386**, 1207-1220 (2022). https://doi.org:10.1056/nejmoa2118691
20 Hatfield, K. M. *et al.* Effectiveness of Coronavirus Disease 2019 (COVID-19) Vaccination Against Severe Acute Respiratory Syndrome Coronavirus 2 (SARS-CoV-2) Infection Among Residents of US Nursing Homes Before and During the Delta Variant Predominance, December 2020-November 2021. *Clin Infect Dis* **75**, S147-S154 (2022). https://doi.org:10.1093/cid/ciac562
21 Powell, A. A. *et al.* Protection against symptomatic infection with delta (B.1.617.2) and omicron (B.1.1.529) BA.1 and BA.2 SARS-CoV-2 variants after previous infection and vaccination in adolescents in England, August, 2021-March, 2022: a national, observational, test-negative, case-control study. *Lancet Infect Dis* **23**, 435-444 (2023). https://doi.org:10.1016/S1473-3099(22)00729-0
22 Grant, R. *et al.* Impact of SARS-CoV-2 Delta variant on incubation, transmission settings and vaccine effectiveness: Results from a nationwide case-control study in France. *Lancet Reg Health Eur* **13**, 100278 (2022). https://doi.org:10.1016/j.lanepe.2021.100278
23 Gazit, S. *et al.* Hybrid immunity against reinfection with SARS-CoV-2 following a previous SARS-CoV-2 infection and single dose of the BNT162b2 vaccine in children and adolescents: a target trial emulation. *Lancet Microbe* **4**, e495-e505 (2023). https://doi.org:10.1016/s2666-5247(23)00103-9
24 Lewis, N. *et al.* Effectiveness Associated With Vaccination After COVID-19 Recovery in Preventing Reinfection. *JAMA Netw Open* **5**, e2223917 (2022). https://doi.org:10.1001/jamanetworkopen.2022.23917
25 Cerqueira-Silva, T. *et al.* Effectiveness of CoronaVac, ChAdOx1 nCoV-19, BNT162b2, and Ad26.COV2.S among individuals with previous SARS-CoV-2 infection in Brazil: a test-negative, case-control study. *Lancet Infect Dis* **22**, 791-801 (2022). https://doi.org:10.1016/s1473-3099(22)00140-2
26 Plumb, I. *et al.* Effectiveness of COVID-19 mRNA Vaccination in Preventing COVID-19–Associated Hospitalization Among Adults with Previous SARS-CoV-2 Infection — United States, June 2021–February 2022. *MMWR Morb Mortal Wkly Rep* **71**, 549-555 (2022). https://doi.org:10.15585/mmwr.mm7115e2
27 Hammerman, A. *et al.* Effectiveness of the BNT162b2 Vaccine after Recovery from Covid-19. *N Engl J Med* **386**, 1221-1229 (2022). https://doi.org:10.1056/nejmoa2119497
28 Khan, F. L. *et al.* Estimated BNT162b2 Vaccine Effectiveness Against Infection With Delta and Omicron Variants Among US Children 5 to 11 Years of Age. *JAMA Netw Open* **5** (2022). https://doi.org:10.1001/jamanetworkopen.2022.46915
29 Carazo, S. *et al.* Estimated Protection of Prior SARS-CoV-2 Infection Against Reinfection With the Omicron Variant Among Messenger RNA–Vaccinated and Nonvaccinated Individuals in Quebec,

Canada. *JAMA Netw Open* **5**, e2236670 (2022). https://doi.org:10.1001/jamanetworkopen.2022.36670
30 Carazo, S. *et al.* Protection against omicron (B.1.1.529) BA.2 reinfection conferred by primary omicron BA.1 or pre-omicron SARS-CoV-2 infection among health-care workers with and without mRNA vaccination: a test-negative case-control study. *Lancet Infect Dis* **23**, 45-55 (2023). https://doi.org:10.1016/s1473-3099(22)00578-3
31 Cerqueira-Silva, T. *et al.* Vaccination plus previous infection: protection during the omicron wave in Brazil. *Lancet Infect Dis* **22**, 945-946 (2022). https://doi.org:10.1016/s1473-3099(22)00288-2
32 Carazo, S. *et al.* Effectiveness of previous infection-induced and vaccine-induced protection against hospitalisation due to omicron BA subvariants in older adults: a test-negative, case-control study in Quebec, Canada. *Lancet Healthy Longev* **4**, e409-e420 (2023). https://doi.org:10.1016/s2666-7568(23)00099-5
33 Yung, C. F. *et al.* BNT162b2 vaccine protection against omicron and effect of previous infection variant and vaccination sequence among children and adolescents in Singapore: a population-based cohort study. *Lancet Child Adolesc Health* **7**, 463-470 (2023). https://doi.org:10.1016/s2352-4642(23)00101-3
34 Shrestha, N. K. *et al.* Coronavirus Disease 2019 Vaccine Boosting in Previously Infected or Vaccinated Individuals. *Clin Infect Dis* **75**, 2169-2177 (2022). https://doi.org:10.1093/cid/ciac327
35 Huang, L. *et al.* Comparing hybrid and regular COVID-19 vaccine-induced immunity against the Omicron epidemic. *npj Vaccines* **7** (2022). https://doi.org:10.1038/s41541-022-00594-7
36 Klein, N. P. Added Benefit of Covid-19 Vaccination after Previous Infection. *N Engl J Med* **386**, 1278-1279 (2022). https://doi.org:10.1056/NEJMe2201380
37 Flacco, M. E. *et al.* COVID-19 vaccines reduce the risk of SARS-CoV-2 reinfection and hospitalization: Meta-analysis. *Front Med* **9**, 1023507 (2022). https://doi.org:10.3389/fmed.2022.1023507
38 Bobrovitz, N. *et al.* Protective effectiveness of previous SARS-CoV-2 infection and hybrid immunity against the omicron variant and severe disease: a systematic review and meta-regression. *Lancet Infect Dis* **23**, 556-567 (2023). https://doi.org:10.1016/s1473-3099(22)00801-5
39 Paul, P. *et al.* Effectiveness of the pre-Omicron COVID-19 vaccines against Omicron in reducing infection, hospitalization, severity, and mortality compared to Delta and other variants: A systematic review. *Hum Vaccin Immunother* **19** (2023). https://doi.org:10.1080/21645515.2023.2167410
40 Buchan, S. A. *et al.* Estimated Effectiveness of COVID-19 Vaccines Against Omicron or Delta Symptomatic Infection and Severe Outcomes. *JAMA Netw Open* **5**, e2232760 (2022). https://doi.org:10.1001/jamanetworkopen.2022.32760
41 Menegale, F. *et al.* Evaluation of Waning of SARS-CoV-2 Vaccine–Induced Immunity. *JAMA Netw Open* **6**, e2310650 (2023). https://doi.org:10.1001/jamanetworkopen.2023.10650
42 Centers for Disease Control and Prevention. *COVID Data Tracker*, <https://covid.cdc.gov/covid-data-tracker> (2023).
43 Samanovic, M. *et al.* Robust immune responses are observed after one dose of BNT162b2 mRNA vaccine dose in SARS-CoV-2–experienced individuals. *Sci Transl Med* **14** (2022). https://doi.org:10.1126/scitranslmed.abi8961
44 Prendecki, M. *et al.* Effect of previous SARS-CoV-2 infection on humoral and T-cell responses to single-dose BNT162b2 vaccine. *Lancet* **397**, 1178-1181 (2021). https://doi.org:10.1016/s0140-6736(21)00502-x
45 Reynolds, C. P., C *et al.* Prior SARS-CoV-2 infection rescues B and T cell responses to variants after first vaccine dose. *Science* **372** (2021). https://doi.org:10.1126/science.abh1282

46 Goel, R. *et al.* Distinct antibody and memory B cell responses in SARS-CoV-2 naïve and recovered individuals after mRNA vaccination. *Sci Immunol* **6** (2021). https://doi.org:10.1126/sciimmunol.abi6950
47 Romero-Pinedo, S. *et al.* Vaccine Type-, Age- and Past Infection-Dependence of the Humoral Response to SARS-CoV-2 Spike S Protein. *Front Immunol* **13** (2022). https://doi.org:10.3389/fimmu.2022.809285
48 Azzi, L. *et al.* Mucosal immune response in BNT162b2 COVID-19 vaccine recipients. *eBioMedicine* **75**, 103788 (2022). https://doi.org:10.1016/j.ebiom.2021.103788
49 Barateau, V. *et al.* Prior SARS-CoV-2 infection enhances and reshapes spike protein–specific memory induced by vaccination. *Sci Transl Med* **15** (2023). https://doi.org:10.1126/scitranslmed.ade0550
50 Efrati, S. *et al.* Safety and humoral responses to BNT162b2 mRNA vaccination of SARS-CoV-2 previously infected and naive populations. *Scientific Reports* **11** (2021). https://doi.org:10.1038/s41598-021-96129-6
51 Lin, D. *et al.* Effectiveness of Bivalent Boosters against Severe Omicron Infection. *N Engl J Med* **388**, 764-766 (2023). https://doi.org:10.1056/nejmc2215471
52 Link-Gelles, R. *et al.* Estimates of Bivalent mRNA Vaccine Durability in Preventing COVID-19–Associated Hospitalization and Critical Illness Among Adults with and Without Immunocompromising Conditions — VISION Network, September 2022–April 2023. *MMWR Morb Mortal Wkly Rep* **72**, 579-588 (2023). https://doi.org:10.15585/mmwr.mm7221a3
53 Hansen, C. H. Evidence of immune imprinting or the effect of selection bias? *Science Advances* **9** (2023). https://doi.org:10.1126/sciadv.adk5668
54 Hamid, S. *et al.* COVID-19–Associated Hospitalizations Among U.S. Infants Aged <6 Months — COVID-NET, 13 States, June 2021–August 2022. *MMWR Morb Mortal Wkly Rep* **71** (2022). https://doi.org:10.15585/mmwr.mm7145a3
55 Evans, R. A. *et al.* Impact of COVID-19 on immunocompromised populations during the Omicron era: insights from the observational population-based INFORM study. *The Lancet Regional Health - Europe*, 100747 (2023). https://doi.org:10.1016/j.lanepe.2023.100747
56 Wiemken, T. *et al.* Seasonal trends in COVID-19 cases, hospitalizations, and mortality in the United States and Europe. *Sci Rep* **13** (2023). https://doi.org:10.1038/s41598-023-31057-1
57 Regan, J. *et al.* Use of Updated COVID-19 Vaccines 2023–2024 Formula for Persons Aged ≥6 Months: Recommendations of the Advisory Committee on Immunization Practices — United States, September 2023. *MMWR Morb Mortal Wkly Rep* **72**, 1140-1146 (2023). https://doi.org:10.15585/mmwr.mm7242e1
